# HMMR potential as a diagnostic and prognostic biomarker of cancer—speculation based on a pan-cancer analysis

**DOI:** 10.3389/fsurg.2022.998598

**Published:** 2023-01-10

**Authors:** Junyi Shang, Xiaoju Zhang, Guangjie Hou, Yong Qi

**Affiliations:** ^1^ Department of Respiratory and Critical Care Medicine; Henan Provincial People's Hospital; People's Hospital of Zhengzhou University, Zhengzhou, China; ^2^ Department of Thoracic Surgery; Henan Provincial People's Hospital; People's Hospital of Zhengzhou University, Zhengzhou, China

**Keywords:** HMMR, pan-cancer, prognosis, immune infiltration, TCGA

## Abstract

**Background:**

Although the status of universal upregulation for the Hyaluronan-Mediated Motility Receptor (HMMR) in pan-cancer is still unknown, HMMR is upregulated and associated with poor prognosis for some tumors.

**Methods:**

Exploring HMMR expression in different tumor types using The Cancer Genome Atlas (TCGA) or other public databases for a pan-cancer analysis, exploring the relationship between HMMR and tumor prognosis, and exploring the role of HMMR in tumor immunity.

**Results:**

No matter the pairing or unpairing of data, HMMR expression generally increased compared to corresponding normal tissue. Based on a CCLE study, our results indicated that HMMR is widely expressed in various tumor cells. For most tumor types, high HMMR expression was associated with reduced Overall Survival (OS), Return to Functional Status (RFS), and Platinum Free Interval (PFI). ROC curves indicated that HMMR displays high prediction potential for most tumor types. In pan-cancer, HMMR is correlated with some clinical staging, immune cells, and immune checkpoints for some tumors. The GO/KEGG enrichment analysis results for proteins most closely related to HMMR indicate that the most highly enriched pathways are all related to tumor development.

**Conclusions:**

Our pan-cancer analysis of HMMR suggests that HMMR can be used as a potential diagnostic and prognostic indicator of pan-cancer and that HMMR may be involved in tumor development.

## Introduction

Cancer occurrence caused by multiple biologic processes is a life-threatening disease and, worldwide, imposes a substantial economic burden (1–3). Each year, tens of thousands of people die of cancer. Due to the lack of adequate diagnosis and therapy, cancer causes a higher mortality rate in less developed countries. Early diagnosis and treatment can significantly reduce cancer morbidity, and effective treatments can increase patient survival rates. Cancer treatments include surgery, chemotherapy, radiation, targeted therapy, immunotherapy, and so forth. Accurate cancer diagnosis requires useful predictive markers. Implementing immune therapy from immune-suppressor checkpoints, such as Programmed Cell Death Protein 1/Programmed Death-Ligand 1 (PD1/PDL1) expression, is often beneficial. Although radical surgery is the most effective treatment, many cancer patients do not benefit from surgery (4). Chemotherapy, radiotherapy, targeted therapies, and immunotherapies do not apply to all patients (5). As such, the discovery and application of new diagnostic markers and therapeutic targets are never out-of-date.

Exposing general phenomena related to cancer has become one of the major hotspots in cancer research. Bioinformatics has made it easier to conduct commonality research related to tumors. One such commonality is the role of the Hyaluronan-Mediated Motility Receptor (HMMR) in breast cancer (6, 7), pancreatic cancer (8), and lung adenocarcinomas (9). HMMR, also called cluster of differentiation 168(CD168), to date, the role of HMMR in pan-cancer has not yet been explored. The purpose of this study was to analyze the expression of HMMR in pan-cancer and its potential value for diagnostic and prognostic use.

## Materials and methods

### The expression of HMMR in pan-cancer

The GTEx dataset was obtained from TCGA (https://portal.gdc.cancer.gov/) and processed through Toil. RNAseq data were downloaded from the HTSeq-FPKM data of TCGA Pan-Cancer. The abbreviations for cancer types are shown in Supplementary Table S1. The RNAseq data of tumors and paracancerous tissues in different cancers also came from the HTSeq-FPKM data of TCGA Pan-Cancer for retention paired samples. The differential expressions of HMMR in GTEx, in paired or unpaired samples from TCGA, were all statistically processed using R (Version 3.6.3) and ggplot2 [Version 3.3.3] for visualization. For analyzing differential HMMR expression between the tumor and adjacent normal tissue, we also used TIMER (https://cistrome.shinyapps.io/timer/) (10). To validate the reliability of TCGA, we used ONCOMINE (https://www.oncomine.org), a public database for retrieving HMMR expression in different cancers. Finally, the HMMR expression matrix for 946 cell lines from 22 types of cancers was obtained from the CCLE (https://portals.broadinstitute.org/ccle/about). To construct HMMR distributions in different tumoral tissues, we used R (v4.0.3) and ggplot2 (v3.3.3).

### The relationship between HMMR and overall survival (Os)

We used the Kaplan-Meier Plotter (https://www.kmplot.com) to analyze the association between HMMR and OS in pan-cancer. Next, prognostic data for 33 types of cancer were downloaded from TCGA. Samples were then distributed into low and high groups based on HMMR expression. The R survivminer package (Version 0.4.9) was used for visualization, and the survival package (Version 3.2–10) was used for statistical analyses of the survival data. The HR and *p* values for each tumor prognosis-based HMMR expression were determined using the Cox regression model, then constructed the forest plots.

### The relationship between HMMR and recurrence free survival (RFS), constructed the forest plot for progress free interval (PFI)

The Kaplan Meier plotter was also used for analyzing the relationship between HMMR and RFS in pan-cancer. We downloaded pan-cancer clinical data from TCGA, divided the data into a high HMMR expression group and a low HMMR expression group, and then obtained the HR and *P* for each type of cancer-based on HMMR expression using a Cox regression model. Statistical analyses and visualization were determined using the R survminer package (Version 0.4.9) and the survival package (Version 3.2–10). We employed forest plots to display the PFI for each tumor type.

### ROC curves based on HMMR expression

RNAseq data were obtained from the TCGA dataset. ROC curves were constructed to predict normal cells or cancer. We used R (Version 3.6.3) for statistical analyses and visualization. The pROC package (Version 1.17.0.1) and the ggplot2 package (Version 3.3.3) were also simultaneously used for analyses, visualization, and for the calculation of the confidence intervals and curve areas.

### The correlation between HMMR and clinical stage

RNAseq and clinical information were obtained from TCGA. Normal and control samples were removed. We retained samples using clinical information. An analysis was implemented for HMMR and the T-stage, with a Kruskal-Wallis test employed for statistical analyses. For statistical analyses and visualization, we utilized R (Version 3.6.3). The program ggplot2 (Version 3.3.3) was also used for visualization.

### The relationship between HMMR and immune infiltration

The RNA-seq data of 33 tumor types were downloaded from the TCGA database. mRNA expression data was also downloaded from 33 tumors with paired normal tissue samples. The immune-related assessment was performed using the immunedeconv package. The package consists of six integrated algorithms, including TIMER, xCell (11), MCP-counter (12), CIBERSORT (13), EPIC (14), and quanTIseq (15). R (v4.0.3) was used for statistical analyses. The significance of the two groups was performed using a Wilcoxon rank sum test.

### The correlation between HMMR and immune checkpoints

SIGLEC15, IDO1, CD274, HAVCR2, PDCD1, CTLA4, LAG3, and PDCD1LG2 are immune checkpoint-related transcripts. We extracted these eight gene expressions of 33 tumor types from TCGA and calculated correlations between HMMR and checkpoint-related transcripts. We used R (Version 4.0.3) for statistical analyses. A rank sum test was used to detect two sets of data. *p*-values ≤ 0.05 were considered statistically significant.

### The correlation between HMMR and tumor mutation burden (TMB) and microsatellite instability (MSI)

Downloaded The RNA-seq database of 33 tumor patients from TCGA, and each tumor matched mRNA expression data Derived TMB from the article by Thorsson et al. (16). and derived MSI from the article by Bonneville et al. (17). Then performed statistical analyses by using R software (Version 4.0.3). A rank sum test was employed on two sets of data. *p* value < 0.05 was considered statistically significant.

### Analysis of molecular correlates

The STRING Database (https://string-db.org/) is a Protein-Protein Interaction (PPI) analysis database that can predict protein-protein interactions of known proteins. Entered HMMR into the STRING website by selecting *Homo sapiens*. Then obtained the top ten HMMR-related proteins and downloaded the results. For constructing the PPI network, we employed R (Version 3.6.3) and the igraph package (Version 1.2.6) for statistical analyses and visualization. Similarly, we obtained the top 50 proteins most tightly associated with HMMR. Then performed enrichment analysis for GO and KEGG terms. The ggplot2 package (Version 3.3.3) and the clusterProfile package (Version 3.14.3) were used for statistical analysis and visualization.

### The protein expression of HMMR in pan-cancer

The Human Protein Atlas (https://www.proteinatlas.org/) is the most extensive collection of immunohistochemistry (IHC) data mapping all human proteins. First, we collected the protein expressions of HMMR in different cancer types and the corresponding normal organs from this website. Selected samples of 5 tumor types to represent the antibody stains in 20 different cancers. HMMR expression is low or not detected in these five cancer types' corresponding normal organs but was the medium or high expression in tumor tissues. The stack bar plot shows the proportion of moderate or increased expression of HMMR in these five tumors; the stack bar plot was plotted by http://www.bioinformatics.com.cn, which is a free online platform for data analysis and visualization. Meanwhile, We collected cancer tissues and paired adjacent normal tissues from eight lung adenocarcinoma patients with pathologic stage I or II. Western blotting detected the HMMR expression, and paired *t*-test was used to analyze the difference between cancer and paired adjacent normal tissues. This study was approved by Henan Provincial People's Hospital ethics committees.

## Results

### Differential HMMR expression in various cancers

**Figure 1:**
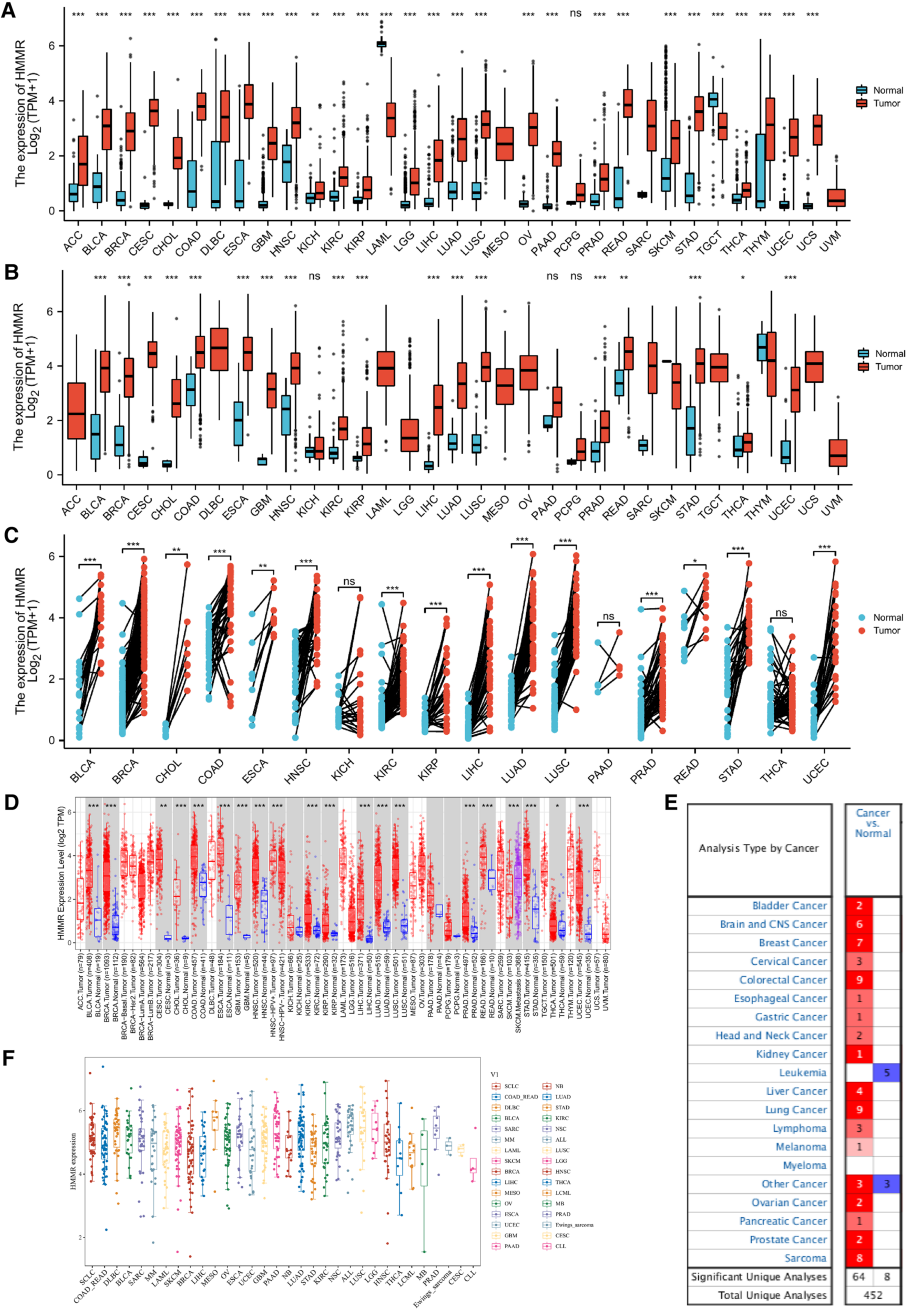
HMMR expression in different types of tumors. The abscissa represents samples in different groups, and the ordinate shows the distribution of HMMR expression. ns, *p* ≥ 0.05; *, *p* < 0.05; **, *p* < 0.01; ***, *p* < 0.001. (**A**) HMMR expression analysis in pan-cancer for GTEx datasets. (**B**) Differential HMMR expression in tumor tissues as compared to normal tissues. (**C**) Different HMMR expressions between the tumor and paired adjacent normal tissues were utilized from the TCGA dataset. (**D**) HMMR expression for the TIMER dataset. (**E**) HMMR expression in different tissues for the ONCOMINE dataset, blue represents low expression, and red represents high expression. (**F**) HMMR expression in different tumor types using the CCLE database.

HMMR mRNA expression levels in ACC, BLCA, BRCA, CESC, CHOL, COAD, DLBC, ESCA, GBM, HNSC, KICH, KIRC, KIRP, LGG, LIHC, LUAD, LUSC, OV, PAAD, PCPG, PRAD, READ, SKCM, STAD, THCA, THYM, UCEC, and UCS were determined to be higher than in normal tissues, except for LAML and TGCT (Figure 1A). A Wilcoxon rank sum unpaired test for TCGA data indicated that the expression of HMMR mRNA in BLCA, BRCA, CESC, CHOL, COAD, ESCA, GBM, HNSC, KIRC, KIRP, LIHC, LUAD, LUSC, PRAD, READ, STAD, THCA, and UCEC was higher than in normal tissue (Figure 1B). For TCGA paired samples, HMMR mRNA expression in BLCA, BRCA, CHOL, ESCA, HNSC, KICH, KIRC, KIRP, LIHC, LUAD, LUSC, PRAD, READ, STAD, and UCEC were all at higher levels in tumor tissues than in matched normal adjacent tissues (Figure 1C).

The expression of HMMR in pan-cancer was also analyzed using the TIMER database. HMMR expression was at a higher level in BLCA, BRCA, CESC, CHOL, COAD, ESCA, GBM, HNSC, KIRC, KIRP, LIHC, LUAD, LUSC, PRAD, READ, SKCM, STAD, THCA, and UCEC as compared to normal tissue (Figure 1D). We also verified differential HMMR expression between tumor and normal tissue using the ONCOMINE software. Except for leukemia and some other cancers, HMMR expression in tumor tissues was significantly higher than in normal tissues (Figure 1E). Finally, we used the Cancer Cell Line Encyclopedia (CCLE) to profile HMMR expression across various types of cancer (Figure 1F).

### The relationship between HMMR expression and overall survival (Os)

**Figure 2:**
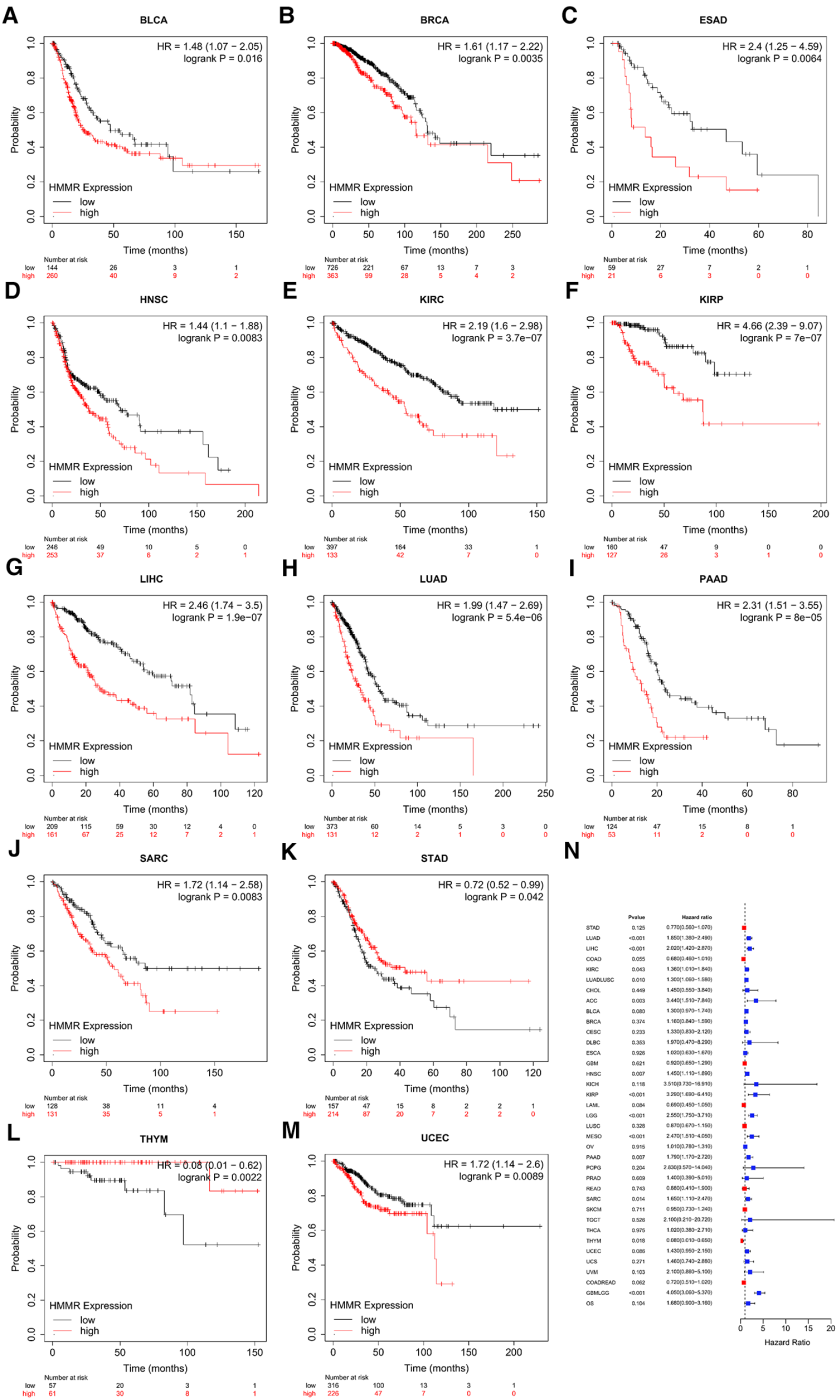
The relationship between HMMR gene expression and OS (**A–M**). (**N**) is the forest plot of HRs for OSs in different tumor types.

Figures 2A–N represents the relationship between HMMR expression and OS across different tumor types. High HMMR expression indicated a reduced OS for bladder carcinoma, breast cancer, esophageal adenocarcinoma, head-neck squamous cell carcinoma, kidney renal clear cell carcinoma, kidney renal papillary cell carcinoma, liver hepatocellular carcinoma, lung adenocarcinoma, pancreatic ductal adenocarcinoma, sarcoma, and stomach adenocarcinoma. For thymoma and uterine corpus endometrial carcinoma, high HMMR expression predicted better OS.

### The relationship of HMMR to RFS and PFI

**Figure 3:**
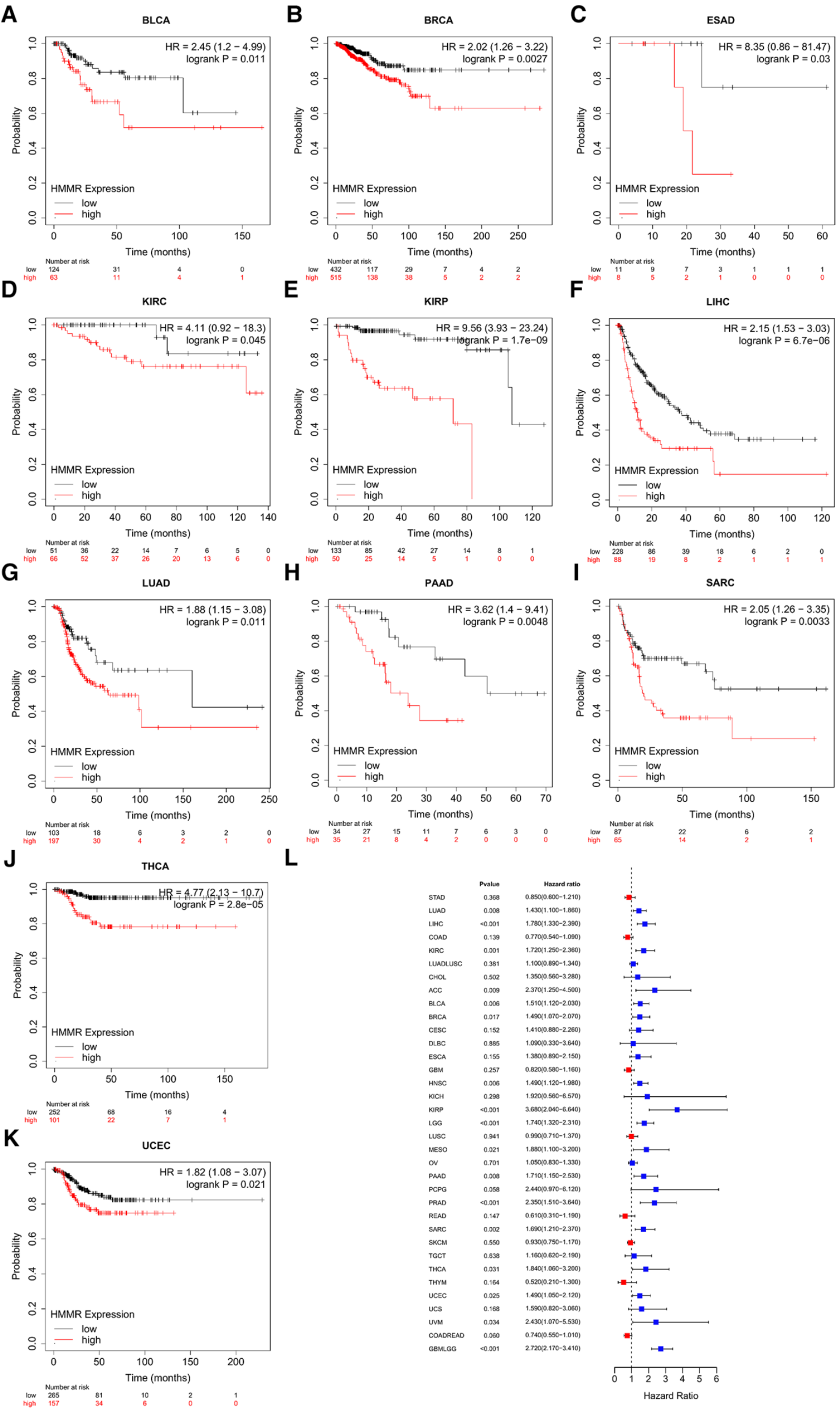
An RFS survival analysis of HMMR expression in pan-cancer (**A–K**). (**L**) is PFI forest plot for HMMR expression in pan-cancer.

For bladder carcinoma, breast cancer, esophageal adenocarcinoma, kidney renal clear cell carcinoma, kidney renal papillary cell carcinoma, liver hepatocellular carcinoma, lung adenocarcinoma, pancreatic ductal adenocarcinoma, sarcoma, thyroid carcinoma and uterine corpus endometrial carcinoma, patients with higher HMMR expression were predicted to have a reduced RFS (Figures 3A–K). Figure 3L shows the correlation between HMMR and PFI for the various tumor types using forest plots.

### ROC curves constructed by HMMR expression

**Figure 4:**
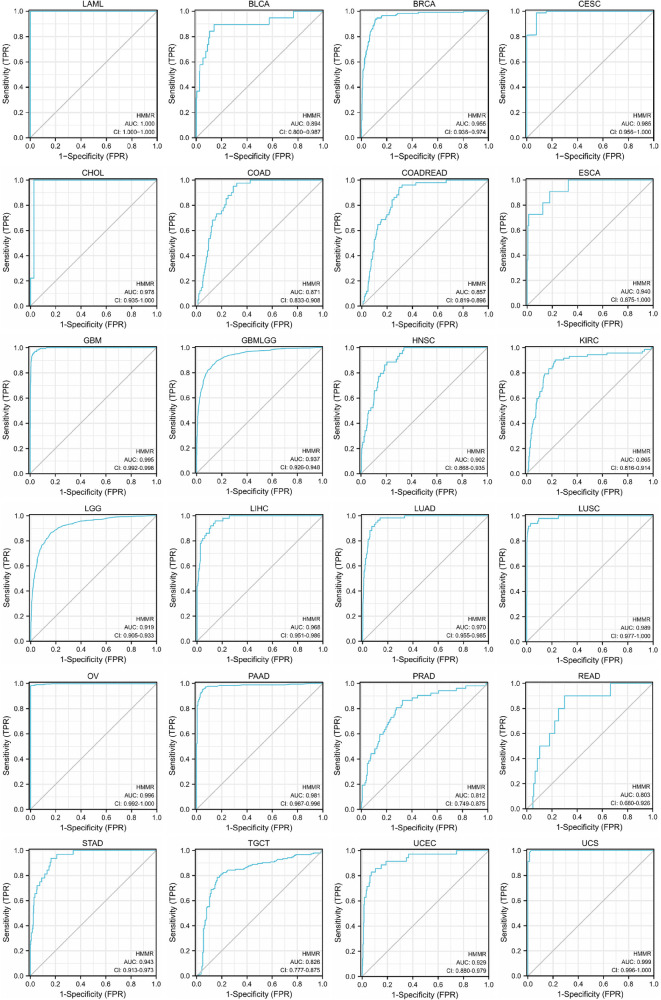
The ROC curve of HMMR predicting tumor and normal outcomes. For Areas Under the ROC Curve (AUC) (which are between 0.5 and 1), the closer the value is to 1, the better the diagnostic value. HMMR displays poor accuracy for AUCs between 0.5 and 0.7. HMMR displays certain accuracy when AUC is between 0.7 and 0.9, and good accuracy when AUC is above 0.9.

For LAML, BRCA, CESC, CHOL, ESCA, GBM, GBMLGG, HNSC, LGG, LIHC, LUAD, LUSC, OV, PAAD, STAD, UCEC, and UCS, HMMR has high accuracy in predicting tumor and normal outcomes. For BLCA, COAD, COADREAD, KIRC, PRAD, READ, and TGCT, HMMR also has a certain accuracy in predicting tumor and normal outcomes (Figure 4).

### The correlation between HMMR expression and tumor stage

**Figure 5:**
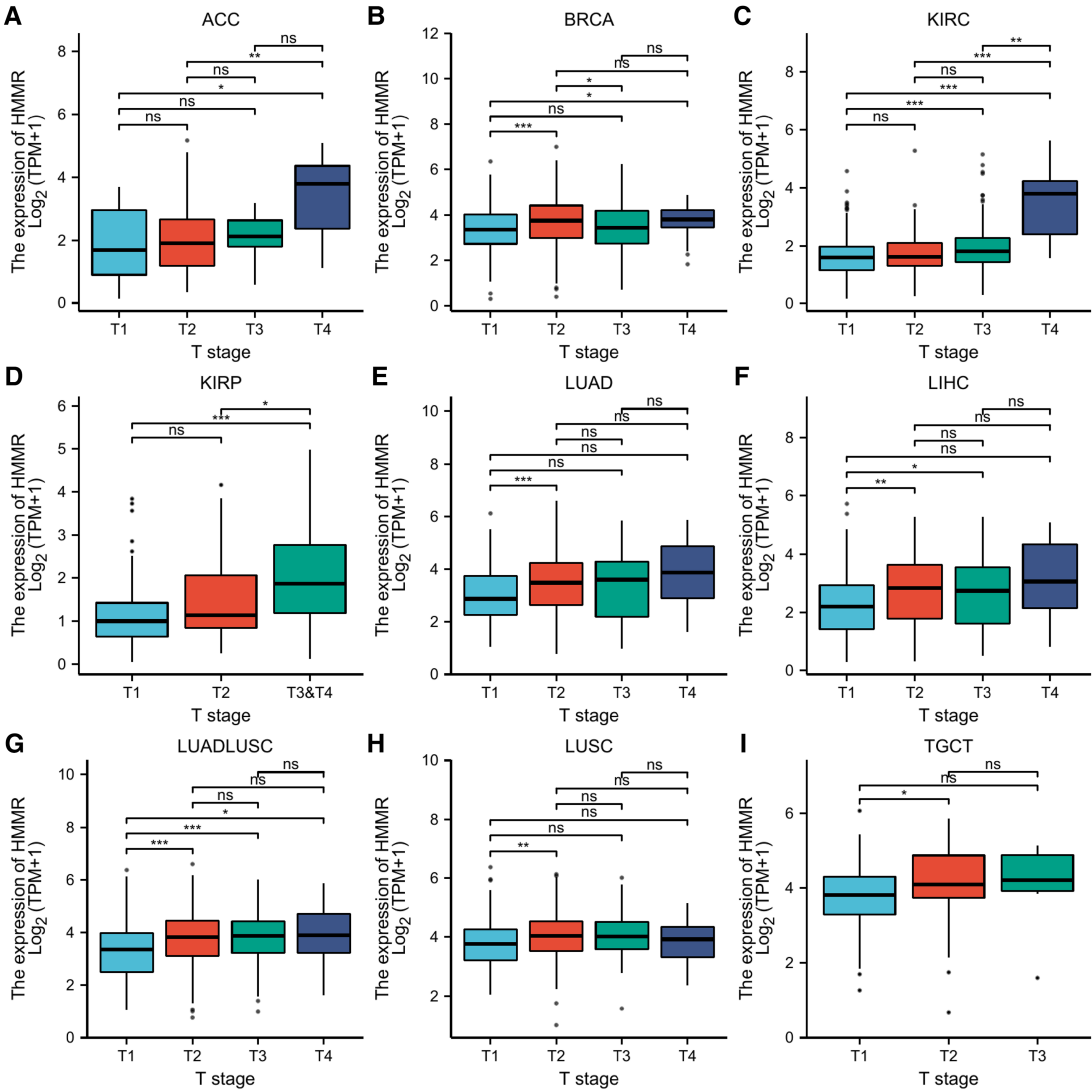
Statistical correlation analysis between HMMR and the pathological T stages. (A) The horizontal axis represents different T stages; the vertical axis represents HMMR expression. ns, *p* ≥ 0.05; *, *p* < 0.05; **, *p* < 0.01; ***, *p* < 0.001.

Based on a correlation analysis between HMMR and different tumor stages, ACC, BRCA, KIRC, KIRP, LUAD, LIHC, LUADLUSC, LUSC, TGCT, and HMMR had certain predictive accuracy for the T stage (Figures 5A–I).

### The correlation analysis between HMMR and immune cells

**Figure 6:**
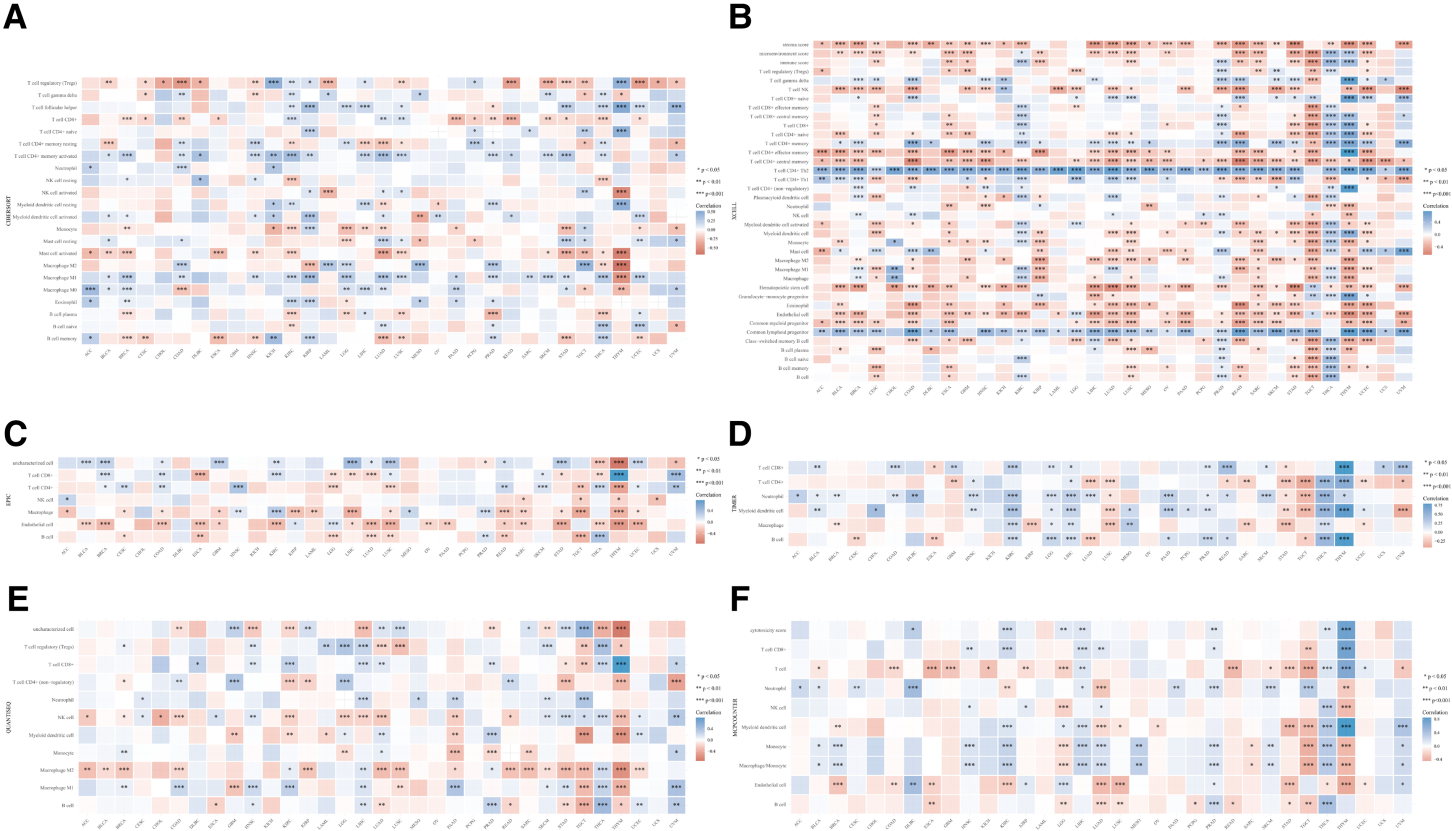
The correlation between HMMR and different immune cells in various tumor types. The horizontal axis represents different tumor types, the vertical axis represents different immune scores, different colors represent the correlation coefficient, negative values indicate an inverse correlation, and positive values indicate a positive correlation. The darker the color, the stronger the correlation. Asterisks represent the degree of importance (*p). *, *p* < 0.05; **, *p* < 0.01; ***, *p* < 0.001. (**A**) CIBERSORT, (**B**) xCell, (**C**) EPIC, (**D**) TIMER, (**E**) quanTIseq, and (**F**) MCP-counter.

Our research separately used CIBERSORT (Figure 6A), xCell (Figure 6B), EPIC (Figure 6C), TIMER (Figure 6D), quanTIseq (Figure 6E), and MCP-counter (Figure 6F) to calculate the correlation between HMMR and immune cells in various tumor types. Figure 6 indicates that HMMR has correlations with multiple different types of immune cells for 33 tumor types (*p* < 0.05).

### Heat maps for the relationship between HMMR expression and immune checkpoint-related genes for various tumor types

**Figure 7:**
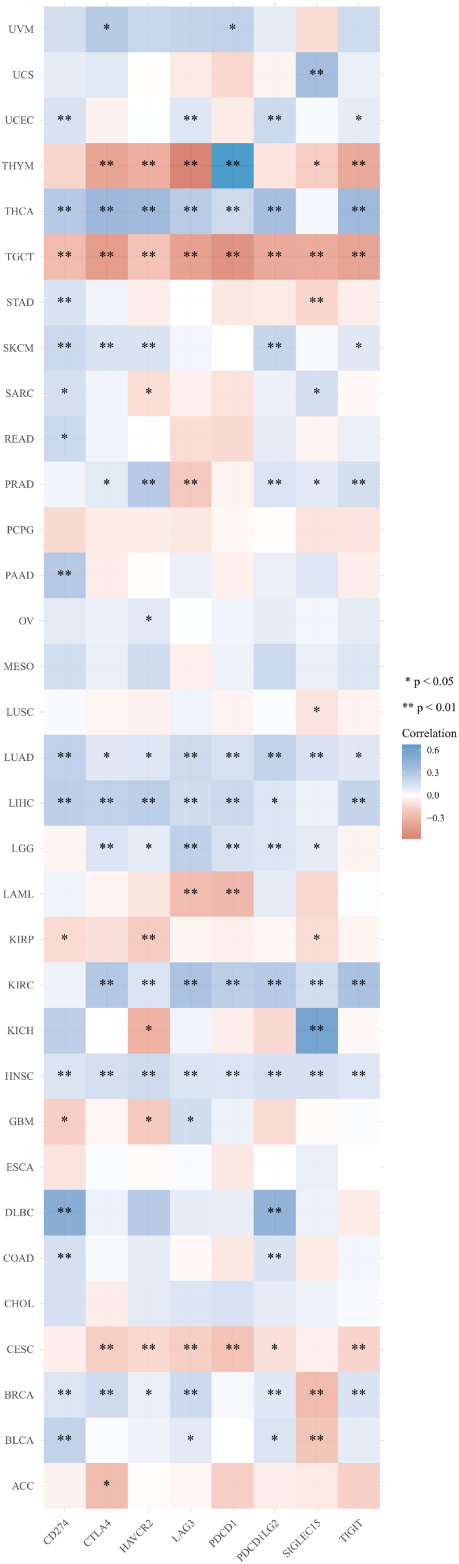
A heat map for the correlation between HMMR and immune checkpoint-related genes in 33 tumor types from TCGA. The horizontal axis provides various immune checkpoint-related genes, and the vertical axis provides various tumor types. Every box in the figure indicates the correlation between HMMR expression and the expression of immune checkpoint-related genes in each tumor. Different colors represent different correlation coefficients. The stronger the color, the stronger the correlation. Asterisks represent the level of importance (*p), *, *p* < 0.05; **, *p* < 0.01, and ***, *p* < 0.001.

SIGLEC15, IDO1, CD274, HAVCR2, PDCD1, CTLA4, LAG3, and PDCD1LG2 are immune checkpoint-related genes. Figure 7 provides a heatmap of the correlation between immune checkpoints and HMMR in 33 tumor types from TCGA. In addition to PCPG, MESO, ESCA, and CHOL, HMMR was associated with multiple immune checkpoint-related genes (*p* < 0.05) (Figure 7).

### The correlation of HMMR with TMB and MSI

**Figure 8:**
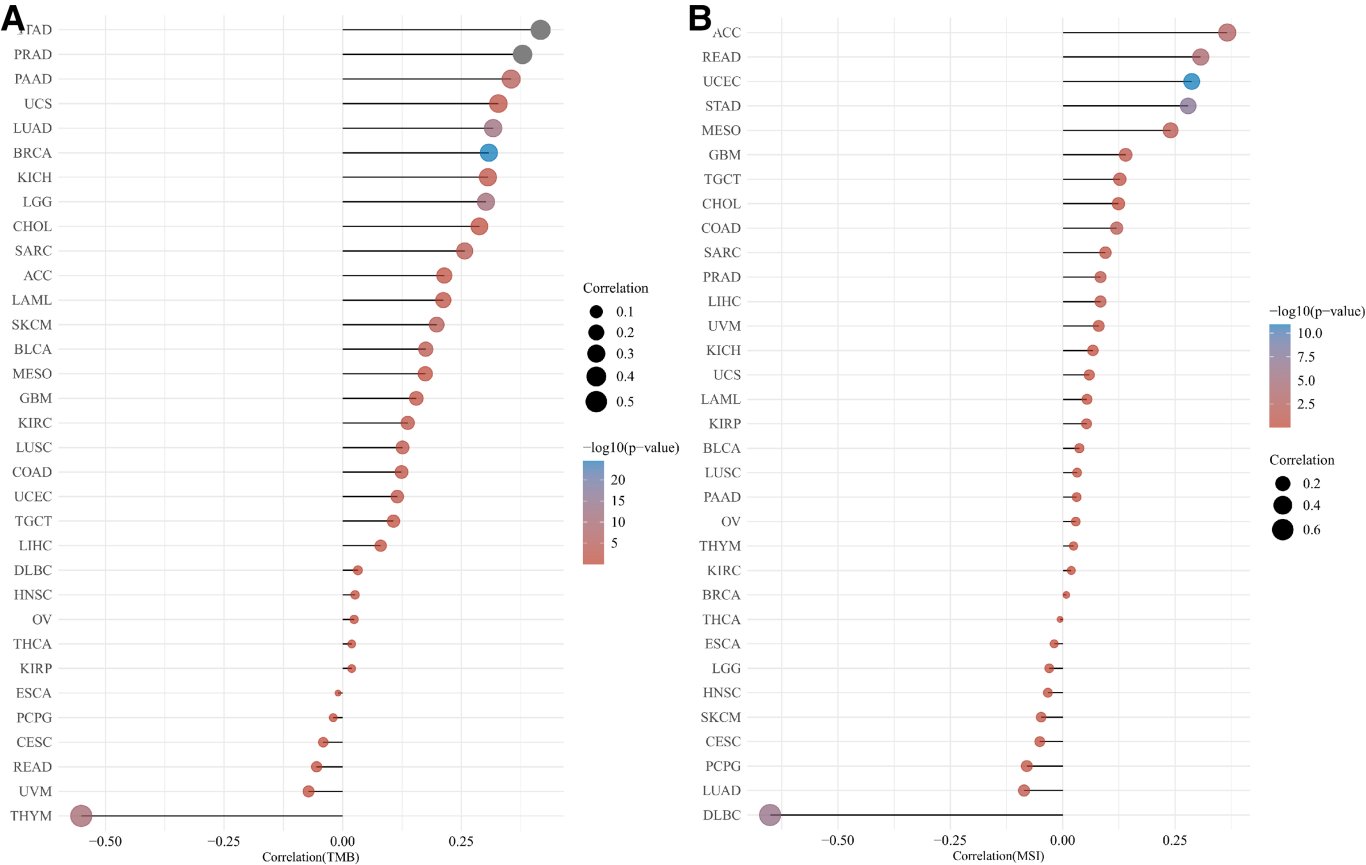
Correlation analysis for HMMR with TMB (**A**) and MSI (**B**) for different tumor types.

Figure 8 provides a Pearson correlation analysis for HMMR expression correlated with TMB (Figure 8A) and MSI (Figure 8B). The abscissa provides the correlation coefficient between the gene and TMB or MSI. The ordinate provides various tumors. Colors represent various *p* values. The darker the blue color, the smaller the *p*-value.

### Molecular correlation analyses

**Figure 9:**
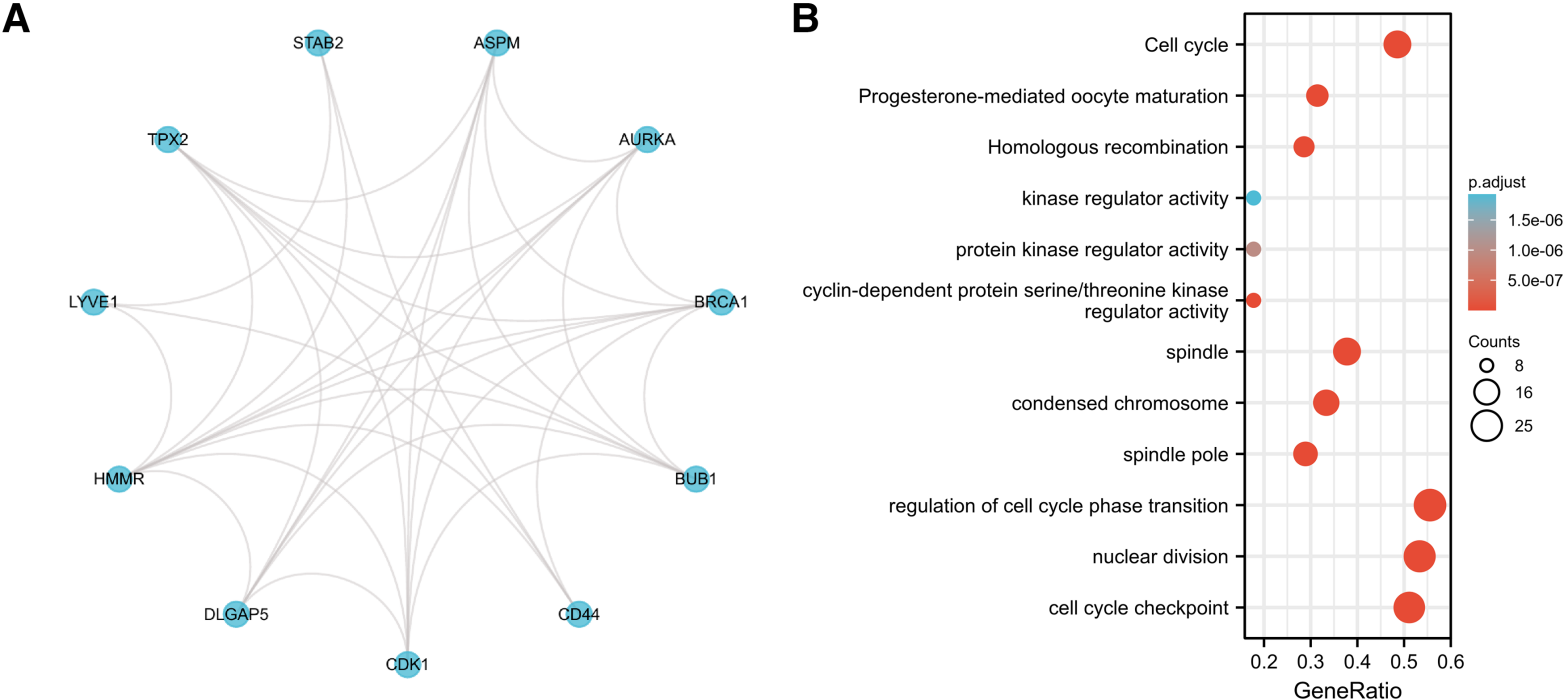
(**A**) the PPI network for the top 10 proteins associated with HMMR. (**B**) A GO/KEGG enrichment analysis for the top 50 proteins associated with HMMR.

We performed a STRING database pathway analysis. The strongest associated proteins to HMMR were LYVE1, TPX2, STAB2, ASPM, AURKA, BRCA1, BUB1, CD44, CDK1, and DLGAP5. The results are provided as a Protein-Protein Interaction (PPI) network (Figure 9A). We then performed a gene GO/KEGG analysis for the top 50 protein associations to HMMR (Figure 9B). Nuclear division and regulation of the cell cycle phase transition were the most enriched GO molecular function term. The most enriched KEGG pathway was the Cell cycle.

### The protein expression of HMMR in pan-cancer

**Figure 10:**
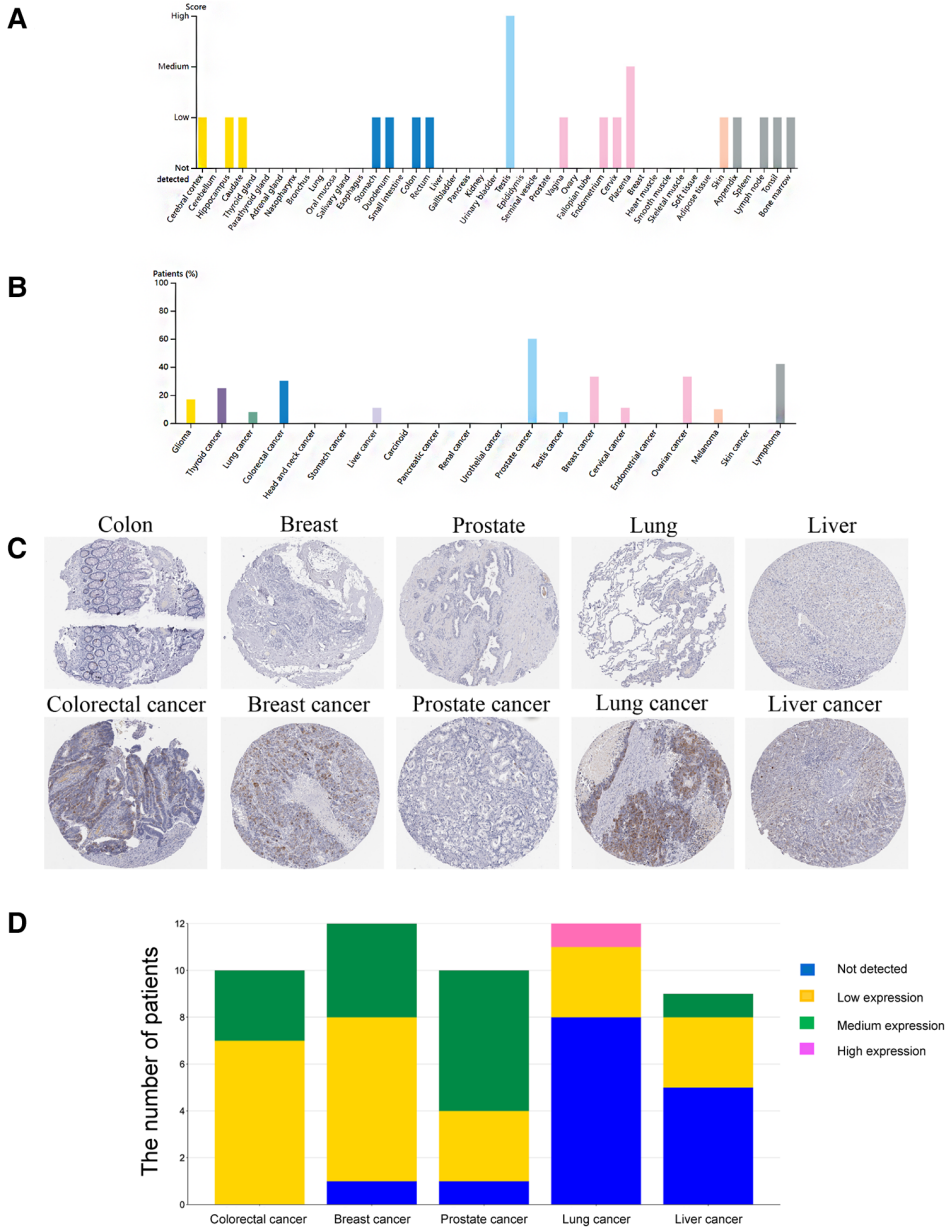
The protein expression of HMMR in different organs and tumors by using the human protein atlas. (**A**) The protein expression score of HMMR in different organs. (**B**) The proportion of patients with medium and high HMMR expression in different tumor types. (**C**) IHC of HMMR expression in five tumor types and their corresponding normal organs. (**D**) The stack bar plot of proportions with different HMMR expressions, blue represents not detected, yellow represents low expression, green represents medium expression, and pink represents high expression.

**Figure 11:**
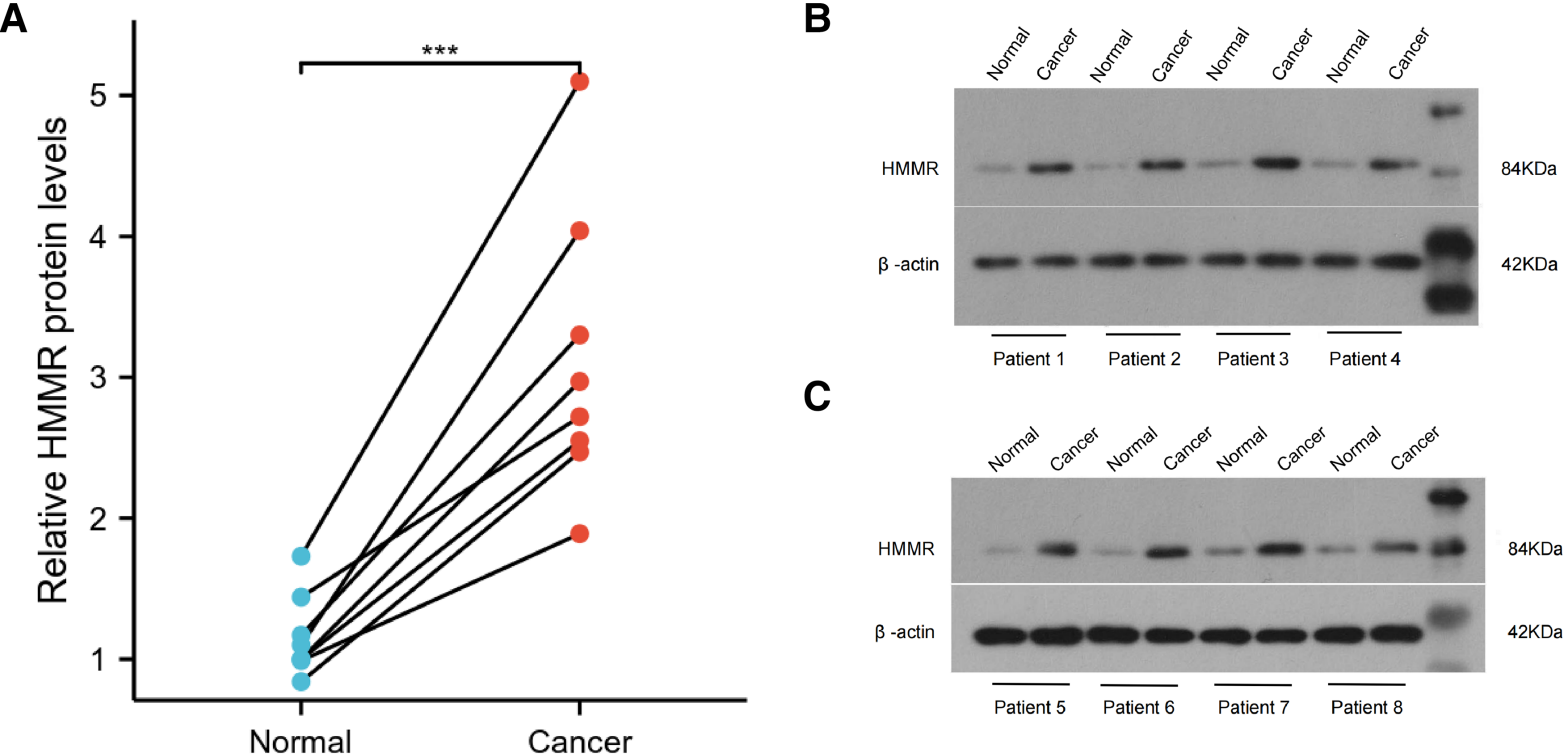
The protein expression of HMMR in lung adenocarcinoma tissues and the cancer-adjacent tissues (A). ***, *p* < 0.001.

IHC detected the expression of HMMR in different organs in The Human Protein Atlas. Except for the medium expressed in the placenta and highly expressed in the testis, HMMR is not detected or has low expression in other organs (Figure 10A). In Figure 10B, we showed the proportion of high or medium expression of HMMR in 20 cancer types. We selected colorectal cancer, breast cancer, prostate cancer, lung cancer, and liver cancer to represent the antibody stains in 20 different cancers; HMMR expression can be detected in these five cancer types while not being detected in their corresponding normal organs (Figures 1C,D). Finally, we validated the results in eight patients with lung adenocarcinoma. HMMR expression was higher in cancer tissues compared with the cancer-adjacent tissues for eight patients with lung adenocarcinoma (Figure 11).

## Discussion

HMMR is a type of hyaluronic acid receptor associated with cell movement (18). HMMR influences brain development by regulating the spindle (19). Elevated HMMR expression is always associated with poor prognosis in breast cancer (20, 21). Some bioinformatics analysis findings have indicated that HMMR is the hub gene for some tumors (22, 23). However, the role of HMMR in all cancer types is largely unknown. The purpose of our study was to explore HMMR in pan-cancer.

HMMR is expressed at low levels in most healthy tissues. Our study of HMMR expression in pan-cancer found that HMMR was upregulated in most tumor types compared to normal tissues. Therefore, HMMR has the potential to be a tumor marker. In order to verify this hypothesis, we separately repeated the analysis using TCGA, TIMER, and ONCOMINE and found the outcomes to be similar.

Tumor heterogeneity includes intratumoral and intertumoral heterogeneity. Tumor tissues are composed of multiple and complex cell types. Currently, the Cancer Cell Line Encyclopedia (CCLE) is the largest tumor cell database (24). As such, a thorough analysis of various tumor cells using the database can reflect the heterogeneity of tumor cells. HMMR was found to be an independent prognostic indicator for prostate cancer (25). However, the prognostic significance of HMMR in pan-cancer is not entirely clear. Based on our prognosis analysis, high HMMR expression predicts inferior survival for bladder carcinoma, breast cancer, esophageal adenocarcinoma, head-neck squamous cell carcinoma, kidney renal clear cell carcinoma, kidney renal papillary cell carcinoma, liver hepatocellular carcinoma, lung adenocarcinoma, pancreatic ductal adenocarcinoma, sarcoma, and stomach adenocarcinoma. Likewise, based on our pan-cancer analysis, a high level of HMMR is associated with inferior RFS and PFI for various tumor types. ROC curves revealed the predictive power of HMMR regarding tumors. When the area under the ROC curve is close to 1, the predictive power of HMMR is more substantial. With the TCGA database analysis, we found that the area under ROC curves was all above 0.9 for most tumor types, indicating that HMMR could be a potential diagnostic marker for these tumors. Although HMMR is predictive in most tumor types, it did not display the exact potential prediction in some tumor types; this illustrates tumor is a heterogeneous disease. Past researchers have outlined HMMR levels that are upregulated in breast cancer and are accompanied by poor pathologic stages and tumor size (26). In our pan-cancer analysis of the tumor stage, we determined associations of HMMR with some tumor stages.

Immunotherapy is an essential hot treatment for cancer therapy and has become the first-line therapy for cancer patients. The essence of immunotherapy is the mobilization of immune cells to kill tumor cells. Changes within the tumor microenvironment inhibit immune cell function and stop immune cells from engulfing tumor cells, thereby, promoting tumor progression and migration. The immune escape of tumors can also suppress or block the immune response. Immunotherapy began in 1983 (27). Targeting immune checkpoint inhibitors is the most common type of immunotherapy, and PD1 and PDL1 are the most commonly used immune checkpoints (28). In recent years, additional potential immune checkpoints have also been determined (29, 30). The immune checkpoints expressed on immune cells belong to a class of immunosuppressive molecules that can regulate the activation of immunity. Immune checkpoint molecules cause the immune system to remain within a normal range; thus, the immune system does not become over-activated. Our research analyses included the correlation between HMMR expression and different types of immune cells. Our results indicate that HMMR could become a new therapeutic target for tumors. With the exception of PD1 and PDL1, Tumor Mutational Burden (TMB) (31) and Microsatellite Instability (MSI) (32) are also commonly used as predictive markers for immunotherapy. We also performed a correlation analysis between HMMR and MSI/MSI.

Studies of molecular interaction are favorable for analyzing molecular mechanisms. The purpose of the PPI network analysis was to explore protein-protein interactions. The STRING database provides a tool for analyzing the interaction between known and predicted proteins. Using a PPI analysis, we obtained the top 10 and top 50 proteins most closely related to HMMR. The imbalance of the cell cycle may cause persistent excessive cell division, resulting in tumorigenesis. GO and KEGG analyses were performed for functional analysis, the largest cluster pathways display the regulation of cell cycle phase transitions, nuclear division, cell cycle checkpoints, cell cycle, spindle, condensed chromosomes, and spindle poles. All of these pathways are closely related to the occurrence and development of cancer (33–35).

Different tumor types share some similar pathophysiology, including gene mutations, immune infiltration, and other aspects. It is now identified tumors with different subtypes or organs have similarities. For example, TP53 mutations can drive multiple tumor types, such as endometrial cancer. But there are also differences manifest in some genetic changes, which shows tumor heterogeneity. Our results showed that HMMR represents different performances among different tumors, prompting us to think that these differ must be viewed separately. The limitations of our study is that restricted by the professionals we engaged, pan-cancer analyses are difficult to obtain simultaneously for many types of tumor specimens. As such, Some of the results could not be verified with our own clinical samples.

Altogether, we performed bioinformatics analysis and came to the conclusion that HMMR is an oncogene for most cancer types.

## Data Availability

The original contributions presented in the study are included in the article/**Supplementary Material**, further inquiries can be directed to the corresponding author/s.
