# Supplementary material for: HMMR potential as a diagnostic and prognostic biomarker of cancer—speculation based on a pan-cancer analysis
Source: Front Surg. 2023 Jan 10;9:998598. doi: 10.3389/fsurg.2022.998598 (PMC9873350; doi:10.3389/fsurg.2022.998598)
Supplement: Supplementary file 1 [file Table1.docx]

| **Full name** | **Abbreviation** | **Full name** | **Abbreviation** |
| --- | --- | --- | --- |
| Adrenocortical carcinoma | ACC | Bladder Urothelial Carcinoma | BLCA |
| Breast invasive carcinoma | BRCA | Cervical squamous cell carcinoma and endocervical adenocarcinoma | CESC |
| Cholangiocarcinoma | CHOL | Colon adenocarcinoma | COAD |
| Colon adenocarcinoma/Rectum adenocarcinoma Esophageal carcinoma | COADREAD | Lymphoid Neoplasm Diffuse Large B-cell Lymphoma | DLBC |
| Esophageal adenocarcinoma | ESAD | Esophageal carcinoma | ESCA |
| Glioblastoma multiforme | GBM | Glioma | GBMLGG |
| Head and Neck squamous cell carcinoma | HNSC | Kidney Chromophobe | KICH |
| Kidney renal clear cell carcinoma | KIRC | Kidney renal papillary cell carcinoma | KIRP |
| Acute Myeloid Leukemia | LAML | Lower Grade GLioma | LGG |
| Liver hepatocellular carcinoma | LIHC | Lung adenocarcinoma | LUAD |
| Lung squamous cell carcinoma | LUSC | Lung adenocarcinoma and squamous cell carcinoma | LUADLUSC |
| Mesothelioma | MESO | Ovarian serous cystadenocarcinoma | OV |
| Pancreatic adenocarcinoma | PAAD | Pheochromocytoma and paraganglioma | PCPG |
| Prostate adenocarcinoma | PRAD | Rectum adenocarcinoma | READ |
| Sarcoma | SARC | Skin cutaneous melanoma | SKCM |
| Stomach adenocarcinoma | STAD | Stomach and Esophageal carcinoma | STES |
| Testicular germ cell tumors | TGCT | Thyroid carcinoma | THCA |
| Thymoma | THYM | Uterine Corpus Endometrial Carcinoma | UCEC |
| Uterine Carcinosarcoma | UCS | Uveal Melanoma | UVM |

**Supplementary Table 1.** The abbreviation of human cancers
